# Revisiting Perdeck's massive avian migration experiments debunks alternative social interpretations

**DOI:** 10.1098/rsbl.2024.0217

**Published:** 2024-07-03

**Authors:** Morrison T. Pot, Marcel E. Visser, Barbara Helm, Jan A. C. von Rönn, Henk P. van der Jeugd

**Affiliations:** ^1^ Department of Animal Ecology, Netherlands Institute of Ecology (NIOO-KNAW), Wageningen, The Netherlands; ^2^ Vogeltrekstation – Dutch Centre for Avian Migration and Demography, Netherlands Institute of Ecology (NIOO-KNAW), Wageningen, The Netherlands; ^3^ Groningen Institute for Evolutionary Life Sciences (GELIFES), University of Groningen, Groningen, The Netherlands; ^4^ Swiss Ornithological Institute, Sempach, Lucerne, Switzerland

**Keywords:** migration, vector navigation, true-goal navigation, social learning, *Sturnus vulgaris*

## Abstract

Whether avian migrants can adapt to their changing world depends on the relative importance of genetic and environmental variation for the timing and direction of migration. In *the* classic series of field experiments on avian migration, A. C. Perdeck discovered that translocated juveniles failed to reach goal areas, whereas translocated adults performed ‘true-goal navigation’. His translocations of > 14 000 common starlings (*Sturnus vulgaris*) suggested that genetic mechanisms guide juveniles into a population-specific direction, i.e. ‘vector navigation’. However, alternative explanations involving social learning after release in juveniles could not be excluded. By adding historical data from translocation sites, data that was unavailable in Perdeck's days, and by integrated analyses including the original data, we could not explain juvenile migrations from possible social information upon release. Despite their highly social behaviour, our findings are consistent with the idea that juvenile starlings follow inherited information and independently reach their winter quarters. Similar to more solitarily migrating songbirds, starlings would require genetic change to adjust the migration route in response to global change.

## 1. Background

Escaping the seasonally deteriorating ecological conditions at the breeding grounds by travelling to more benign wintering areas presents juvenile migratory birds with the unconceivable challenge of accurate navigation to yet unknown destinations [1,2]. In songbirds, the general view of *how* the first migration is performed has settled around the idea of inborn ‘vector navigation’: an inherited programme describing the migratory route as a number of predisposed flights into a specific compass direction, where the length and duration of flights are controlled by endogenous circannual rhythms [3,4]. Later in life, i.e. after completion of the first autumn migration, inherited spatiotemporal instructions are complemented with learned site-specific information such as landmarks, geomagnetic parameters and olfactory information, which allows for more accurate navigation to familiar destinations [5–8].

Whether inherited instructions *alone* can explain migrations of juvenile songbirds is currently under debate as the evidence provided so far does not exclude social cues that, depending on the species, may provide important additional navigational information [1,9–13]. Direct evidence for vector navigation informed by inherited programmes is claimed from two different experimental approaches. Firstly, field experiments based on ring recoveries and tracking showed that juveniles translocated to unfamiliar areas continued autumn migration in their population-specific direction (but see [13,14]), thereby missing the population-specific winter quarters [15,16]. Importantly, simultaneously released adults did correct for the displacement. This age difference was seen to underline the importance of inheritance prior to acquiring experience. The second approach comprised experiments with captive songbirds, where preferred take-off directions were quantified in the simplified and confined environment of, for instance, an orientation cage [17], showing that juveniles express directional preferences that are correlated to the migratory behaviour of their populations of origin [18–20]. Recently, cutting-edge studies combining genomics and individual tracking of songbirds showed evidence for the genetic inheritance of the migratory direction [21,22].

Potential effects of the social environment, however, are notoriously difficult to investigate in translocation experiments [23]. Because studies reporting on translocation experiments usually lack knowledge about the migratory behaviour of the receiving local populations, it remains often unclear whether the expressed migratory behaviour of translocated individuals results from inherited instructions or from phenotypic adjustments by copying the behaviour of local conspecifics. Considering social cues as a potentially important source of navigational information during the first migration [11,24], requires us to rethink results from early translocation experiments with free-flying songbirds that lead to the idea of inborn vector navigation in the first place [25,26].

**Figure 1.**
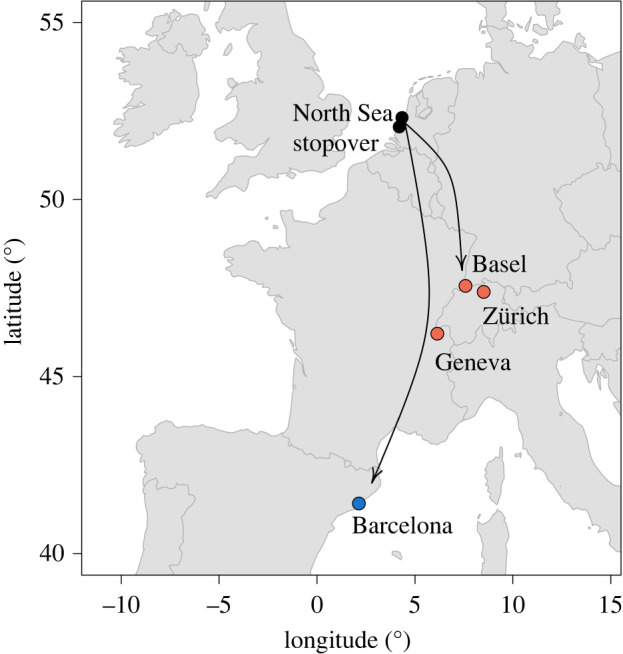
Overview of Perdeck's experiments. For both ‘the Switzerland experiment’ (red) and ‘the Spain experiment’ (blue), thousands of starlings were translocated from autumn stopovers along the Dutch North Sea coast to Switzerland (1948–1957) and Spain (1959–1962).

Here, we leverage our unique access to two sources of historical data to re-assess the importance of social cues in juvenile migrants by revisiting *the* classic series of field experiments in the study of avian migration by A. C. Perdeck. In massive translocation experiments he investigated migratory mechanisms in the common starling (*Sturnus vulgaris*) (hereafter: ‘starling’), a short-distance migratory songbird. By translocating > 14 000 migrating starlings from an autumn stopover area in The Netherlands to Switzerland [15] and Spain [27,28], ontogenetic aspects of orientation were investigated (figure 1). Perdeck released translocated birds either separated by age or in mixed-age groups. The obtained ring recoveries indicated that only translocated adults adjusted their migratory direction towards their northwest European wintering grounds, and thus performed ‘true-goal navigation’, i.e. the ability to re-locate a familiar goal from an unfamiliar area [29]. Translocated juveniles, in contrast, were recovered west to southwest of the release sites, suggesting that these maintained their migratory direction, i.e. vector navigation [29], and thereby failed to reach the population-specific wintering grounds. However, starlings are highly social animals and the translocated juveniles migrated in a direction that is typical for many starling populations. An in-depth review [12], following earlier suggestions [20,30–33], discussed that Perdeck's results may just as well provide evidence for the importance of social cues from local conspecifics. Perdeck [15] himself acknowledged this limitation in the study design: ‘*The high sociability of migrating starlings raises the question whether the birds did join the populations that normally migrate over the area in question. Reliable data about the preferred direction of these populations are lacking*’. Whether inherited instructions are overruled by social cues is important to understand, because copying the behaviour of others is often associated with rapid adjustment to anthropogenic change [33,34].

## 2. Methods

Perdeck's original records are stored in a paper archive at the Dutch Centre for Avian Migration and Demography and were digitized for the purpose of these analyses. We obtained recoveries of starlings that were captured at the bird ringing sites ‘Loosduinen’ (52°05′ N, 4°20′ E) and ‘Wassenaar’ (52°31, 4°34′ E) located in the coastal dunes of the southwest Netherlands. This dataset included 101 recoveries that were obtained from 3588 adults and 258 recoveries from 11 247 juveniles that were translocated by airplane to Basel, Zürich and Geneva (Switzerland) in October to November 1948–1957 (hereafter: ‘the Switzerland experiment’), 31 recoveries from 885 adults and 130 recoveries from 2703 juveniles that were translocated by airplane to Barcelona (Spain) in October to November 1959–1962 (hereafter: ‘the Spain experiment’), and 392 recoveries from *ca* 7500 juveniles that were released in The Netherlands during both experiments (controls). A selection of juveniles was translocated to Switzerland in mixed-age groups containing an equal number of adults or in single-age groups. Group compositions were reconstructed using the translocation date and destination. All birds were released within 24 h after capture. For details, see [15,27].

Next, we obtained recoveries from the Swiss and Catalan Ornithological Institutes to compare the migratory directions of starlings that were translocated by Perdeck with local conspecifics. Data available in the institutional databases and the EURING databank [35] were complemented with 1500 historical recoveries of starlings ringed in Switzerland that were stored on punched cards, which were digitized for these analyses. Owing to variation in the availability of data, we estimated the migratory directions of local Swiss starlings using subsets of data. First, inspections of recoveries indicated that the local breeding population was to some extent still present in Switzerland when Perdeck released his experimental birds. Hence, we used recoveries of starlings that were ringed during the breeding seasons (Apr–Jun) of 1948–1957 (years in which translocations were performed) and were recovered during the subsequent autumn or winter. Secondly, we selected recoveries of starlings that were ringed during *any* autumn in Switzerland (range = 1926–2014) and recovered within that same autumn or the subsequent winter. Recoveries of local Spanish starlings were hardly available for the experimental years. We therefore estimated their migratory behaviour using recoveries of starlings that were ringed in Spain during *any* autumn (range = 1950–2018) and recovered during that same autumn or subsequent winter.

We always used recoveries obtained within the same autumn (Oct–Nov) of ringing and the subsequent winter (Dec–Jan) and discarded recoveries for which the date of ringing or recovery was indicated to be accurate to > 1 week. We only selected recoveries of controls when captured and ringed on dates when translocations took place to construct control groups for the translocation experiments that are not confounded by factors linked to capture date. Owing to a limited number of within-season recoveries, we complemented controls for the Spain experiment with within-season recoveries obtained at other bird ringing sites in the Dutch coastal dunes. Following Perdeck [15], we assumed only individuals recovered at > 50 km distance from their release site to have continued migrating. This final dataset consisted of 506 recoveries (for sample sizes per subset of data, see electronic supplementary material, table S1).

### (a) Statistical analyses

Using the Rayleigh test of uniformity, we tested whether directions between release and recovery locations in the different experimental groups differed significantly from a unimodal distribution [36,37] and rejected the null hypothesis for most groups: only autumn recoveries of experimental birds in Spain and all recoveries of starlings naturally migrating through Spain were randomly distributed (electronic supplementary material, table S1). We interpreted random distributions as non-migratory movements. We analysed migratory directions using multivariate analysis of variance (MANOVA), an approach recently shown to be a powerful method for multi-factorial modelling of circular data [38,39]. We used the *x* and *y* component of the migratory directions respective of the geographic north as a response variable by calculating the sine and cosine of the migratory direction in radians [39,40]. Owing to variation in the availability of data across age classes and treatments (e.g. no adult recoveries in controls), we created several separate models.

First, using a subset of 189 recoveries of translocated individuals, we tested for an age-effect on the migratory direction following translocation while accounting for the season of recovery by including ‘age at capture’, ‘season of recovery’ (Oct–Nov: autumn, Dec–Jan: winter) and their interaction term (age × season of recovery) as fixed factors. By entering ‘experiment’ (Switzerland/Spain) and the two interaction terms (age × experiment and season × experiment), we tested for confounding effects of the two experiments included in the dataset (electronic supplementary material, table S2). Secondly, to test for an effect of translocation on the migratory direction of juveniles, i.e. differences between control and experimental groups, we used a subset of 347 recoveries of juveniles that were captured in the Netherlands and either released locally or translocated. We entered ‘treatment’ (translocation: yes/no), ‘season of recovery’ and their interaction term (translocation × season of recovery) as fixed factors. By entering ‘experiment’ and the two interaction terms (treatment × experiment and season × experiment), we tested for confounding effects (electronic supplementary material, table S3). Thirdly, to test for an effect of group composition (mixed-age groups: juveniles translocated with an equal number of adults; single-age groups: juveniles only) on the migratory direction of translocated juveniles, we used a subset of 122 recoveries of juveniles translocated to Switzerland for which group composition was known and entered ‘group composition’, ‘season of recovery’ and their interaction term (group composition × season of recovery) as fixed factors (electronic supplementary material, table S4). Lastly, to test whether the migratory direction of translocated juveniles differed from local Swiss and Spanish starlings, we created separate models for the two experiments using subsets of 362 and 39 recoveries, respectively, and entered ‘treatment’ (translocation/local), ‘season of recovery’ and their interaction term (treatment × season of recovery) as fixed factors (electronic supplementary material, table S5–S7).

All analyses were performed in R version 4.1.3 [41] using the *deg.dist* and *earth.bear* functions in the package ‘fossil’ [42] to calculate compass bearings in degrees and distances in kilometres between release and recovery locations, and the package ‘circular’ for analyses of directional data [40,43].

## 3. Results

**Figure 2.**
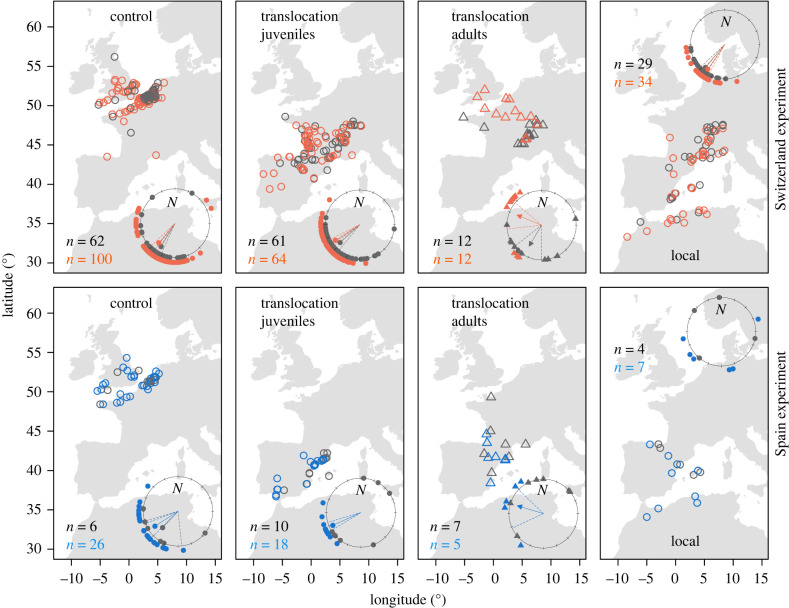
Within-season recoveries of juvenile (○) and adult (△) starlings in populations relevant for Perdeck's translocation experiments. Shown are autumn (grey) and winter (red/blue) recoveries of starlings that were captured along the Dutch North Sea coast during autumn and released locally (controls) or in Switzerland/Spain (translocations), and starlings captured in the translocated areas (locals). Control datasets differ in terms of years in which individuals were captured (1948–1957 and 1959–1962, respectively). Insets show directions in degrees between release and recovery sites. Arrows show the mean group directions (relative to the magnetic north) and the length of each arrow indicates a measure of directedness (*r*). Dotted lines show 95% confidence intervals (only for groups expressing a unimodal distribution). *N*-values refer to the number of autumn and winter recoveries shown in insets (only recoveries obtained at > 50 km from the release sites are shown in insets).

We re-assessed the robustness of Perdeck's conclusion that only adults corrected for translocation. Our analysis of only the original data showed that control juveniles made a short-distance migration due west to southwest (Switzerland experiment, mean direction *α* = 220°, 95% confidence interval (CI) 213–227°, *n* = 100, Rayleigh test of uniformity, *r* = 0.81, *p* < 0.001; Spain experiment, *α* = 237°, *n* = 26, Rayleigh test of uniformity, *r* = 0.86, *p* < 0.001), towards wintering grounds in The Netherlands, Belgium, France and the British Isles (figure 2: controls). An age effect on the mean migratory direction expressed by translocated individuals (MANOVA, *F* = 31.22, *p* < 0.001), was consistent with the conclusion of Perdeck that only translocated adults adjusted by migrating due northwest, towards wintering grounds indicated by controls, i.e. areas where the translocated adults had presumably wintered a year earlier (figure 2: translocations, adults).

The mean migratory direction of translocated adults was affected by the time of year in which they were recovered (MANOVA, age × season of recovery: *F* = 5.36, *p* < 0.01). In the Switzerland experiment, adults shifted from an initially southwest migratory direction when recovered in autumn (*α* = 211°, 95% CI 180–232°, *n* = 12, Rayleigh test of uniformity, *r* = 0.71, *p* = 0.001) to northwest when recovered in winter (*α* = 293°, 95% CI 263–310°, *n* = 12, Rayleigh test of uniformity, *r* = 0.78, *p* < 0.001). This suggests that it took translocated adults up to several weeks before selecting a final migratory destination. Similar effects were present in the Spain experiment, as adult recoveries obtained during autumn were not significantly oriented (Rayleigh test of uniformity, *r* = 0.56, *p* = 0.11), whereas recoveries obtained during winter showed a significant orientation due northwest (*α* = 287°, 95% CI 245–314°, *n* = 5, Rayleigh test of uniformity, *r* = 0.80, *p* = 0.03). Translocated adults initially continued in the population-specific direction before adjusting towards their northwest European goal area (figure 2: translocations, autumn versus winter recoveries).

Translocated juveniles migrated to wintering grounds in a west to south-westerly mean direction from the Swiss and Spanish release sites (Switzerland experiment, *α* = 237°, 95% CI 231–243°, *n* = 64, Rayleigh test of uniformity, *r* = 0.91, *p* < 0.001; Spain experiment, *α* = 246°, 95% CI 239–254°, *n* = 18, *p* < 0.001). They expressed a more westerly direction than controls (electronic supplementary material, table S1), which is likely explained by the latter being guided by the North Sea coastline (figure 2: controls versus translocations, juveniles). Their mean migratory direction was affected by the time of year in which they were recovered (MANOVA, *F* = 7.99, *p* < 0.001), but varied among experiments (experiment × season of recovery, MANOVA, *F* = 10.17, *p* < 0.001), with those juveniles translocated to Spain initially expressing a random distribution (autumn recoveries, *n* = 10, Rayleigh test of uniformity, *r* = 0.17, *p* = 0.77). In a subset of juvenile recoveries with known group composition (*n* = 146), there was no evidence for an effect of group composition (translocated with or without an equal number of adults) on the mean migratory direction (MANOVA, *F* = 1.44, *p* = 0.24). In fact, none of the obtained recoveries suggested that juveniles followed adults after translocation as all juveniles migrated due southwest of the release sites, towards an area covering southern France and northern Spain (figure 2: translocations, juveniles).

We obtained additional ring recoveries from populations migrating through the areas of release in Switzerland and Spain, data that were not available at the time when Perdeck analysed his data, but which we retrieved from institutional archives. We asked whether age-specific responses to translocation can be alternatively explained by translocated juvenile starlings responding to social cues from local conspecifics encountered after release. Juveniles that were translocated to Switzerland and recovered during winter expressed migratory directions that differed significantly from those ringed in Switzerland during the autumn migration period (MANOVA, *F* = 12.76, *p* < 0.001) and the breeding season (MANOVA, *F* = 9.09, *p* < 0.001), as both Swiss-ringed groups migrated in a more southerly mean direction (autumn, *α* = 215°, 95% CI 209–221°, *n* = 34, Rayleigh test of uniformity, *r* = 0.95, *p* < 0.001; breeding, *α* = 213°, 95% CI 207–219°, *n* = 16, Rayleigh test of uniformity, *r* = 0.97, *p* < 0.001) towards the Mediterranean (figure 2: locals, electronic supplementary material, figure S1). In addition, the local populations regularly crossed the Mediterranean Sea to destinations in North Africa, a pattern that was absent in the translocated juveniles (figure 2, electronic supplementary material, figure S1). There was also no evidence that juveniles translocated to Spain adjusted their migratory direction to that of the local autumn population. The local Spanish starlings expressed a random distribution (Rayleigh test of uniformity, *r* = 0.59, *p* = 0.09), suggesting residency (figure 2: locals). By contrast, juveniles translocated to Spain were unimodally recovered south-westerly, indicating migration (figure 2). Thus, although several authors, including Perdeck himself, have suggested that the translocated juveniles may have phenotypically adjusted by joining flocks of local starlings migrating through the areas of release, we can clearly falsify this alternative explanation.

## 4. Discussion

Given their pronounced social behaviour, it seems intuitive to assume that starlings would use social cues to increase their navigation accuracy, especially during the first migration. We cannot exclude the possibility of translocated individuals temporarily joining flocks of local conspecifics (including adults; figure 2) to gain benefits such as thermal energy transfer, safety from predators and information about feeding grounds [44]. However, our data debunk the possibility that juveniles copied the distances and directions of conspecifics encountered in the social environment of the translocation sites. These results may be partly explained by recent tracking data showing that for actual migration flights, starlings resorted to nocturnality, and may thereby be more lonesome travellers than previously thought [45]. Although nocturnality does not necessarily exclude social behaviour, we suggest that this is unlikely since the majority of nocturnally migrating birds travel solo [46], and to the best of our knowledge, starlings do not produce nocturnal flight calls like, for instance, American wood warblers (*Parulidae*) [47] and European thrushes (*Turdidae*) [48]. Our analysis suggests that migratory mechanisms in starlings are not different from other songbirds, in which migratory behaviour is largely genetically predisposed.

Our findings contribute to the current debate on whether songbirds adjust to conspecifics encountered during migration. They suggest that Perdeck's experiments provide support for juvenile starlings mainly following a navigational vector instead of conspecifics. Whether this vector itself solely results from an inherited migratory programme or whether learning is involved remains unclear. The first migration may be viewed as a developmental process in which inherited spatiotemporal instructions are complemented with experiential learning from interactions with the environment [12,14,49]. Exploiting the ever-ongoing development of tracking techniques that would allow for large-scale tracking across age classes [50], in combination with experimental studies such as carried out by Perdeck now over 70 years ago, will further illuminate the extent to which individual differences in migratory behaviour results from genetic, ecological and social variation, and how these factors interact. Populations of migrant species are heavily affected by climatic warming and changes in land use [51,52]. In our starlings, innovating new migratory routes would therefore be a relatively slow process that requires genetic change [53,54]. Insights as presented here are crucial for understanding whether and how fast migrants can adapt to current rates of global change.

## Data Availability

The data are provided in electronic supplementary material [55].
